# Examining the role of urgency in predicting binge size in bulimia nervosa

**DOI:** 10.3389/fpsyg.2023.1166119

**Published:** 2023-05-31

**Authors:** Heather A. Davis, Gregory T. Smith

**Affiliations:** ^1^Department of Psychology, Virginia Tech, Blacksburg, VA, United States; ^2^Department of Psychology, University of Kentucky, Lexington, KY, United States

**Keywords:** bulimia nervosa, *ad lib* test meal, positive urgency, positive affect, binge eating, personality

## Abstract

Greater binge size within bulimia nervosa is associated with elevated distress and impairment. Theoretical models posit that emotion dysregulation predicts binge eating, but little research has investigated the potential for dispositional traits that reflect difficulty in emotion regulation to predict binge size among women with bulimia nervosa. Research supports that negative urgency, the tendency to act rashly when feeling distressed, is associated with binge eating behavior among individuals with bulimia nervosa. Relatively fewer studies have explored associations between binge eating and positive urgency, the tendency to act rashly when feeling extreme positive affect. The urgency traits may predict greater binge size within bulimia nervosa. The current study sought to examine negative urgency and positive urgency as predictors of test meal intake in a sample of 50 women, *n* = 21 with bulimia nervosa and *n* = 29 healthy controls. Dispositional levels of positive urgency, negative urgency, positive affect, and negative affect were measured prior to a laboratory binge eating paradigm. Participants in the bulimia nervosa group scored higher on negative urgency, positive urgency, and negative affect than participants in the control group. Across participants, lower levels of negative affect were associated with greater test meal intake. Elevated levels of positive urgency predicted significantly greater test meal intake, but only for participants with bulimia nervosa. No other dispositional traits predicted test meal intake when the interaction of positive urgency and group was included in the model. Findings suggest positive urgency is an underappreciated, but potentially important, risk factor for greater binge size in bulimia nervosa.

## Introduction

Binge eating (consuming an objectively large amount of food while feeling a loss of control) is a core component of bulimia nervosa (BN). Although loss of control may be more important than the amount of food consumed in understanding distress related to binge eating (Latner et al., 2007), people with BN have recurrent objectively large binge eating episodes by definition (APA, 2013), and consideration of binge size may help inform understanding of BN (Mourilhe et al., 2021).

Accumulating evidence suggests binge size is an important marker of distress and impairment, particularly within BN. A recent meta-analysis found that caloric intake during binge eating episodes was positively correlated with depressive symptoms in BN (Mourilhe et al., 2021). Greater binge size also is associated with lower quality of life, higher weight/shape concerns, elevated anxiety symptoms, greater weight suppression, and more eating disorder impairment (Forney et al., 2016; Keel et al., 2019; Li et al., 2019; Bruzas et al., 2022). As such, further investigation into the predictors of binge size is warranted. Dispositional traits may be associated with greater binge size. If so, such traits may be targeted in BN interventions.

One set of dispositional traits that merit consideration are negative urgency (NU) and positive urgency (PU). NU refers to the tendency to engage in rash behavior when experiencing negative affect (Cyders and Smith, 2008). PU is characterized by engaging in rash action when experiencing positive affect (Cyders and Smith, 2007; Cyders and Smith, 2008). Both are part of a family of five impulsigenic traits that contribute to impulsive behavior, but are only modestly correlated with each other (Whiteside and Lynam, 2001; Cyders and Smith, 2007; Smith et al., 2007). Multiple meta-analyses have established that, of the impulsigenic traits, NU and PU consistently demonstrate the largest correlations and effect sizes with many forms of impulsive behaviors, including bulimic behaviors, substance use, and suicidality (Fischer et al., 2008; Coskunpinar et al., 2013; Berg et al., 2015; Smith and Cyders, 2016). NU has been widely investigated as a risk factor for and correlate of bulimic pathology. Across studies, NU predicts the development of binge eating among adolescents (Pearson et al., 2012; Davis and Smith, 2018), is elevated in BN compared to control groups (Fischer et al., 2008) and is associated with binge eating frequency cross-sectionally and longitudinally (Fischer et al., 2018; Kenny et al., 2019). However, the association between NU and binge size has been less studied.

Although PU has been studied relatively less in relation to eating pathology than NU, and some research suggests PU may not be relevant to eating pathology (Cyders and Smith, 2007), three recent studies support potential associations between PU and binge-spectrum eating disorders. In a clinical sample, PU (but no other impulsigenic trait) was associated with non-suicidal self-injury in BN (Claes et al., 2015). In another clinical sample, PU was cross-sectionally associated with binge frequency among binge-spectrum eating disorders (Michael and Juarascio, 2021). A third study found participants with binge eating disorder reported elevated levels of PU and NU, compared to overweight and non-overweight controls, and the two traits were associated with elevated eating pathology (Kenny et al., 2019). Altogether, findings support examining the potential role of PU in BN symptom expression, including binge size.

Broadly, emotion regulation theories posit that individuals with BN attempt to manage intense affect (negative or positive–but more research focuses on negative) by engaging in binge eating (Pearson et al., 2015; Safer, 2015). Accordingly, NU and PU may prompt binge eating in BN. Emotional intensity is thought to decrease following binge eating, thus providing negative reinforcement (Pearson et al., 2015; Safer, 2015). Importantly, NU and PU each correlate with emotion dysregulation in cross-sectional and longitudinal studies (King et al., 2018; Feil et al., 2020), suggesting they reflect dispositional challenges with responding to affect.

Despite the historical focus on negative emotion regulation (Pearson et al., 2015) and findings demonstrating decreases in positive affect prior to binge eating (Schaefer et al., 2020), there is emerging concern regarding the potentially important role of positive emotion dysregulation in eating disorders (Selby et al., 2019). Theory underlying associations between PU and binge eating is limited, but it may be that individuals high in PU experience positive, rather than negative, reinforcement from binge eating, given high levels of dispositional positive affect (e.g., excitement) and the inherent rewarding properties of palatable foods often consumed while binge eating (de Macedo et al., 2016). For example, eating a large amount of cake at a party may provide positive reinforcement in the form of mood enhancement. Indeed, theory posits rash action while feeling strong positive emotions provides positive reinforcement by enhancing a celebratory mood (Cyders and Smith, 2008) and positive affect has been shown to increase following binge eating (Wonderlich et al., 2018; Schaefer et al., 2020). Further, individuals who binge eat report eating when they feel especially good (Johnson et al., 1995). In contrast, individuals higher in NU (versus PU) may be more prone to negative reinforcement from binge eating.

It is thus possible that elevations in NU and PU contribute to not only risk for binge eating (Davis and Smith, 2018) but also binge size. Few studies have tested associations between urgency and binge size. Forney et al. (2016) found that NU was not associated with binge size among women with BN, but they did not examine potential associations with PU. Also, because binge size in this study was self-reported, it is unclear if or how NU may predict binge size measured objectively.

The current investigation sought to evaluate preliminary associations between the urgency traits and binge size using a laboratory *ad lib* test meal investigation. Because binge eating has been modeled in the laboratory when certain instructions are provided and individuals with BN indicate their laboratory behavior mirrors their binge eating elsewhere, laboratory episodes can provide an acceptable analog to real-life binge eating (Mitchell et al., 1998; Sysko et al., 2018). To our knowledge, no previous study has examined NU or PU in relation to binge size in the laboratory. Based on previous research (Fischer et al., 2008; Kenny et al., 2019), we sought to replicate the finding that women with BN report higher levels of NU and PU than controls. Our core hypothesis was that NU and PU would predict greater caloric consumption (larger binge size), during the *ad lib* test meal for women with BN, but not controls. Given high conceptual and empirical overlap between NU and PU (Cyders and Smith, 2007) we included both in the same model. Because NU and PU correlate with negative and positive affect, respectively, and higher negative affect is associated with binge eating risk (Smith et al., 2020) we included both in the model to control for the potential influence of dispositional tendencies to feel negative or positive affect in the absence of urgency.

## Methods

### Participants

Data come from a larger study of affective consequences of binge eating (Davis et al., 2022). Women (*N* = 50)^1^ were recruited from a large, public university in the southeastern United States and the community to fill two groups: a BN group that met DSM-5 criteria for BN^2^ (*N* = 21) and a healthy control group (*N* = 29). Eligibility criteria were modeled after previous *ad lib* test meal studies (Keel et al., 2018). Advertisements on social media and in the community invited normal-weight women with no eating concerns and women who binge ate and used weight control methods to contact the research team. Eligibility criteria were verified by telephone screen conducted by a research assistant under the supervision of a licensed psychologist. Bulimic behavior questions from the Eating Disorder Examination (Cooper and Fairburn, 1987) were used to confirm study group. Participants were required to be 18–25 years old and have a body mass index (BMI) between 18.5–26.5, based on self-report and verified with objective measure.^3^

Exclusion criteria for all participants included current medical conditions or medication use that could influence appetite, weight, or ability to safely participate, current pregnancy, or lactation. Exclusion criteria for the BN group included being in or seeking eating disorder treatment and having a lifetime history of anorexia nervosa. Control group exclusion criteria included lifetime history of eating disorder symptoms, dietary restriction within the past 8 weeks, and excessive exercise. Women with BN were required to endorse consuming at least 1,000 kcal during binge eating episodes with a loss of control. Calories consumed were calculated by a trained research assistant, using nutritional information available on the internet (Keel et al., 2018).

Participant mean (SD) age was 19.44 (1.55) years. Participants self-identified as 74% White/European American, 10% Black/African American, 8% Hispanic, 6% Asian/Pacific Islander, and 2% biracial. BN participants were significantly older than control participants [*t*(*df*) = 3.67 (48), *p* < 0.001; mean difference of 1.46 years]. Groups did not differ significantly on race, ethnicity, household income, religion, or BMI (all *p*s > 0.05).

### Measures

Food consumption. *Ad lib* test meal food was weighed before and after using a food scale. Intake was calculated in grams consumed by taking the difference of the two values and then converted to calories using the gram to calorie ratio of the food.

Urgency. The UPPS-P Impulsive Behavior Scale (Lynam et al., 2006) measured PU and NU prior to the test meal. The PU subscale consists of 14 items and the NU subscale consists of 12 items. Items are rated on a 4-point Likert scale anchored at (1) Agree Strongly and (4) Disagree Strongly. An example PU item is “I tend to act without thinking when I am really excited.” An example NU item is “When I am upset, I often act without thinking.” Subscale items are averaged for a mean score. The UPPS-P urgency scales demonstrate good reliability and validity (Cyders and Smith, 2007). Internal consistency in the current study was high (NU α =0.92; PU α = 0.94).

Dispositional Affect. The Positive and Negative Affect Schedule (PANAS; Watson et al., 1988) measured dispositional negative and positive affect prior to the test meal. The PANAS consists of 20 words that describe different emotions. Participants indicated to what extent they generally felt each word using a 5-point Likert-scale anchored at (1) Very Slightly or Not at All and (5) Extremely. The negative affect scale includes 10 negative emotions; an example is “distressed.” The positive affect scale includes 10 positive emotions; an example is “proud.” Items are summed for each scale. The PANAS demonstrates high test–retest reliability and criterion validity (DePaoli and Sweeney, 2000; Serafini et al., 2016). Internal consistency in the present study was high (negative affect α = 0.89; positive affect α = 0.92).

#### Procedures

The study was approved by the Institutional Review Board (IRB) at the University of Kentucky (protocol #43524). Written informed consent was obtained. All ethical guidelines in the treatment of study participants and in analyzing the retrieved data were followed. Procedures included one study visit that lasted up to two hours.

Participants were provided a 200-kcal standardized breakfast (store-bought yogurt with pre-measured granola) to consume between 9 am and 9:30 am the day of their study visit. They were instructed not to consume anything else until their 2 pm study visit (Kissileff et al., 1996). When they arrived, participants were asked if they followed the instructions, to ensure they remained eligible. If still eligible, height and weight were measured and a pregnancy test was administered. Participants completed questionnaires on an iPad.

The *ad lib* test meal included three foods requested by participants (Raymond et al., 2007). Participants with BN were asked to report three store-bought foods they typically consumed when binge eating, and controls were asked to report three store-bought foods they enjoyed eating. Common examples of requested food were pretzels and cookies. Enough food was provided to allow for objectively large consumption: 2000 calories of each food item was placed into a bowl or plate and served at an individual place-setting (Wolfe et al., 2002). Participants also were provided an 8-ounce bottle of water. Participants were presented with the test meal and instructions were provided verbally: “Let yourself go and eat as much as you can” (Kissileff et al., 1996). Participants were left alone in the room with the food. Immediately upon finishing the test meal, participants completed questionnaires (results reported elsewhere; Davis et al., 2022).

Participants were debriefed on the purpose of the research, provided compensation in cash ($30) for community-recruited participants or course credit for student participants, and provided a mental health resource list.

#### Data analysis

Data were analyzed using SPSS v.28. Descriptive statistics included means for test meal consumption and scores on UPPS-P PU, UPPS-P NU, PANAS positive affect, and PANAS negative affect. Independent samples *t*-tests were used to compare groups on study variables.

Hierarchical linear regression was used to assess prediction of test meal consumption from group membership, UPPS-P NU and PU, PANAS traits, and the interactions of personality traits with group membership. We constructed the interaction terms as follows. Diagnostic group (BN or control) was dummy coded, with control group as the reference. PU, NU, positive affect, and negative affect were mean-centered. We then calculated the product term of group by each of the personality variables. We were then able to determine if the interaction term had incremental predictive power above and beyond the main effects for each variable. Test meal intake was specified as the outcome variable. At step one, we entered group, PU, NU, positive affect, negative affect, and age (to control for group differences in age). At step two, we entered the interaction terms: group by PU, group by NU, group by positive affect, and group by negative affect.

## Results

### Descriptive statistics

Table 1 shows descriptive statistics for test meal consumption and scores on urgency and affect by group. Table 2 presents correlations among calories consumed during test meal, NU, PU, negative affect, positive affect, and age. Women with BN consumed significantly more calories during the test meal compared with controls. In line with our first hypothesis, women with BN scored significantly higher on NU and PU compared with controls. Women with BN scored higher on negative affect, but not positive affect, compared with controls. Among the BN group, significant positive correlations were observed between total calories consumed and PU. NU and PU were positively correlated across all participants.

**Table 1:** Descriptive statistics for all study variables.

Variable	Mean (*SD*)		*t*	*d*
	Bulimia Nervosa (*n* = 21)	Healthy Control (*n* = 29)		
Total calories consumed during *ad lib* test meal	1276.35 (629.22)	554.06 (235.60)	5.67***	1.63
UPPS-P Negative Urgency	2.80 (0.58)	1.75 (0.54)	6.54***	1.87
UPPS-P Positive Urgency	2.06 (0.74)	1.48 (0.51)	3.28**	0.91
PANAS Negative Affect	22.38 (6.22)	14.72 (4.89)	4.87***	1.37
PANAS Positive Affect	32.71 (8.57)	35.34 (8.53)	1.07	0.31

Total *N* = 50. PANAS, Positive Affect Negative Affect Schedule. ***p < 0.001, **p < 0.01. *d* = 0.20 represents a small effect size, *d* = 0.50 represents a medium effect size, *d* ≥ 0.80 represents a large effect size.

**Table 2:** Correlations among all study variables.

	Total Calories Consumed	UPPS-P Negative Urgency	UPPS-P Positive Urgency	PANAS Negative Affect	PANAS Positive Affect	Age
Total calories consumed	–	0.16	0.49*	−0.23	0.01	0.43
UPPS-P Negative Urgency	−0.02	–	0.61**	0.41	−0.35	−0.19
UPPS-P Positive Urgency	−0.22	0.70***	–	0.32	0.01	0.28
PANAS Negative Affect	−0.19	0.35	0.30	–	−0.17	0.01
PANAS Positive Affect	0.14	−0.49**	−0.49**	0.01	–	0.12
Age	−0.18	0.43*	0.18	0.32	0.10	–

*N* = 50. Shaded region represents bulimia nervosa group. Unshaded region represents control group. PANAS, Positive Affect Negative Affect Schedule. **p < 0.01, *p < 0.05, ***p < 0.001.

### Prediction of *ad lib* test meal intake

**Figure 1:**
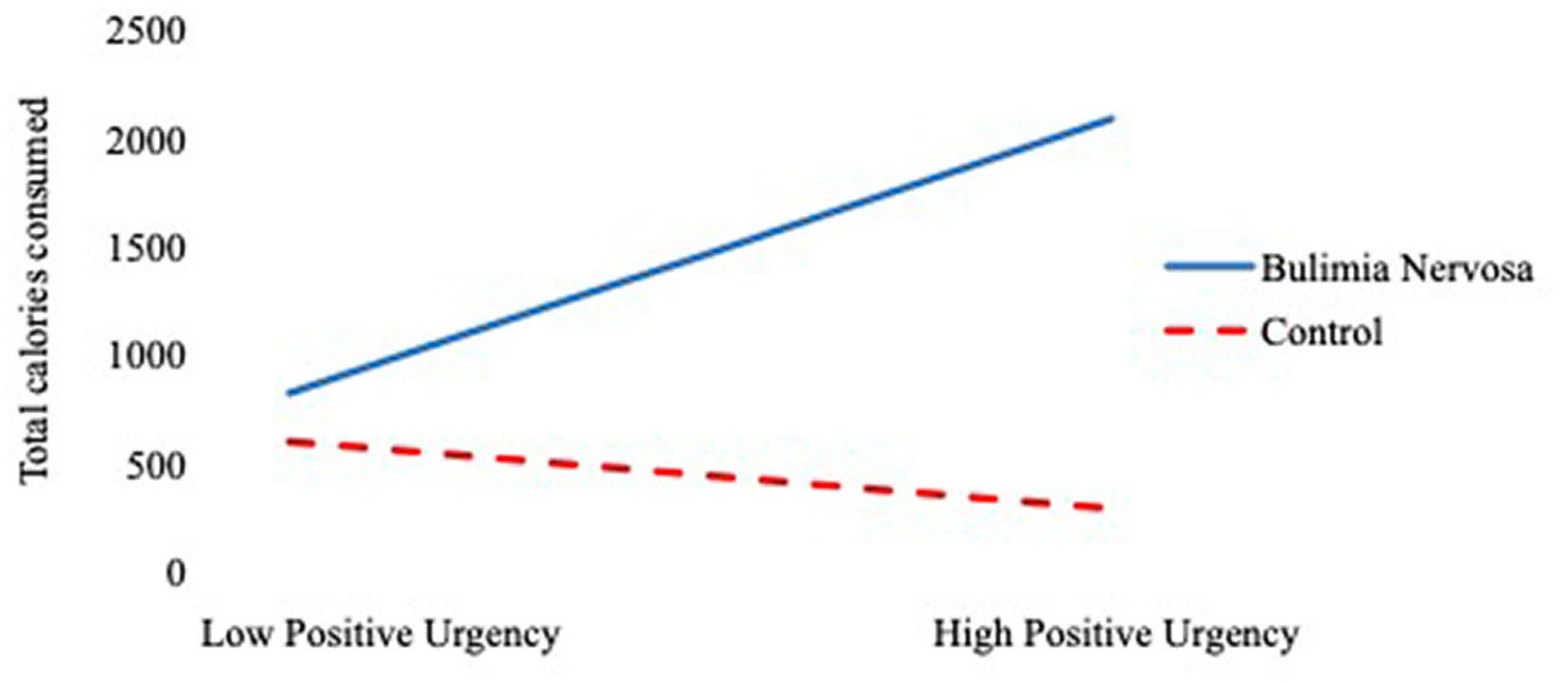
The regression of total calories consumed on positive urgency as a function of group membership.

Given observed correlations among study variables, we checked collinearity in the regression model by measuring variance inflation factor (VIF) values. All VIF values were under 10, indicating no problems with collinearity (Fox, 1991). Table 3 presents the regression results for the prediction of test meal intake from age, group, personality variables, and their interactions. At step 1, only group and negative affect significantly predicted *ad lib* test meal intake. Specifically, BN group membership predicted greater test meal intake. Negative affect level negatively predicted test meal intake across participants. There was no main effect of age, PU, NU, or positive affect on *ad lib* test meal intake. At Step 2, the main effect of group and the interaction of PU by group significantly predicted *ad lib* test meal intake.^4^ The main effects of age, PU, NU, positive affect, negative affect, and the interaction terms between group and NU, positive affect, and negative affect were not significant at Step 2. As Figure 1 shows, among participants with BN, as PU increased, caloric intake during the *ad lib* test meal increased. Among participants in the control group, there was not a significant relationship between PU and caloric intake during the *ad lib* test meal. The change in *R*^2^ from Step 1 to Step 2 was not significant, indicating no increase in incremental predictive power.

**Table 3:** Hierarchical linear regression model predicting food consumption in *ad lib* test meal.

Predictor	*B* (*SE*)	*β*	*t*	*p*	*R*^2^	*F* Change
Step 1						
Age	74.42 (44.57)	0.20	1.67	0.10	0.49	8.72***
Group	674.86 (179.21)	0.59	3.77	<0.001		
UPPS-P Negative Urgency	22.02 (153.69)	0.03	0.14	0.89		
UPPS-P Positive Urgency	249.55 (129.34)	0.30	1.93	0.06		
PANAS Negative Affect	−28.19 (11.73)	−0.33	−2.40	0.02		
PANAS Positive Affect	4.82 (7.74)	0.07	0.62	0.54		
Step 2						
Age	54.13 (47.61)	0.15	1.14	0.26	0.55	2.46
Group	706.90 (177.79)	0.62	3.98	<0.001		
UPPS-P Negative Urgency	94.93 (206.65)	0.13	0.46	0.65		
UPPS-P Positive Urgency	−140.66 (205.12)	−0.17	−0.69	0.50		
PANAS Negative Affect	−12.06 (16.39)	−0.14	−0.74	0.47		
PANAS Positive Affect	2.05 (10.55)	0.03	0.20	0.85		
UPPS-P Negative Urgency X Group	−130.74 (326.72)	−0.11	−0.40	0.69		
UPPS-P Positive Urgency X Group	661.77 (271.00)	0.58	2.44	0.02		
PANAS Negative Affect X Group	−31.61 (22.31)	−0.25	−1.42	0.16		
PANAS Positive Affect X Group	−9.13 (15.52)	−0.09	−0.59	0.56		

*N* = 50. PANAS, Positive Affect Negative Affect Schedule. ***p < 0.001.

## Discussion

The goal of the current preliminary study was to evaluate dispositional levels of NU and PU in the prediction of binge size among women with BN compared with controls. As expected, women with BN reported higher levels of NU and PU, compared with controls. Hypotheses were partially supported: PU predicted greater *ad lib* test meal intake among women with BN but not control women. Contrary to our hypothesis, NU was not associated with *ad lib* test meal intake in women with BN compared to control women. Dispositional levels of broad negative and positive affect also were not significant predictors of test meal intake.

Although prior work has demonstrated cross-sectional associations between PU and self-reported eating disorder symptoms (Claes et al., 2015; Kenny et al., 2019; Michael and Juarascio, 2021), this investigation is the first to support an association between PU and objectively measured binge size among women with BN in a controlled laboratory study. It is striking that PU was the only significant predictor of binge size, above and beyond NU and dispositional levels of negative and positive affect. Results suggest the proneness to act rashly when feeling extreme positive emotions may be uniquely associated with greater binge size in BN. Binge eating episodes frequently include highly palatable foods rated as favorable and rewarding (de Macedo et al., 2016). PU correlates with reward responsiveness (Smith et al., 2023) and predicts other impulsive behaviors perceived as rewarding (e.g., substance use, gambling (Clark et al., 2019; Tanabe et al., 2019)). During the *ad lib* test meal, we provided participants with BN foods they typically consumed during binge episodes, which were frequently highly palatable (e.g., cookies). It is possible that those with BN and high PU were more likely to find these foods rewarding, and thus more likely to consume a greater amount of food when instructed to binge.

It is surprising that NU did not emerge as a significant predictor of objectively measured binge size in the current study, given associations between BN and NU in prior research (Fischer et al., 2008, 2018). Notably, this result aligns with previous work finding no association between NU and self-reported binge size in BN (Forney et al., 2016). NU may play a central role in the development and maintenance of binge eating behavior (Pearson et al., 2012; Davis and Smith, 2018), but a less prominent role in binge size.

When the interaction of group and PU was not included in the model, higher levels of negative affect were associated with lower caloric intake across participants. Negative affect has been associated with lower calorie intake, which may explain this finding (Moreno-Domínguez et al., 2012). However, it is noteworthy that negative affect was no longer significant when the interaction of group and PU was included in the model. This finding lends support to the notion that it is not just a propensity toward feeling negative or positive emotions that predicts binge size, but perhaps the combination of propensity to experience greater levels of positive affect combined with the tendency to act rashly when experiencing intense emotion.

The literature on binge eating and BN has focused primarily on negative emotion dysregulation. As such, theory regarding positive emotion dysregulation, and, consequently, PU, is underdeveloped (Selby et al., 2019). The current findings indicate PU may be important in the risk process for binge eating. It may be that those with high levels of PU receive positive reinforcement (rather than negative reinforcement) from binge eating. This may be related to elevations in reward sensitivity observed in BN (Harrison et al., 2010). Indeed, women with BN show increased response to eating even when satiated, compared to controls (Bischoff-Grethe et al., 2021), which may contribute to overeating. Increased attention to positive reinforcement would diverge from the historical focus on negative emotion regulation, but may help inform understanding of high PU in BN.

Future studies also may investigate the potential for PU to increase risk for or maintain other forms of eating pathology. Previous work has found PU is also cross-sectionally associated with problematic exercise (Kotbagi et al., 2017). Because PU is associated with impaired decision making (Kräplin et al., 2014) and individuals with binge eating prefer sooner, smaller rewards rather than delayed, larger rewards (Brassard and Balodis, 2021), an individual with BN and high PU may be prompted to binge eat for the short-term positively reinforcing qualities of food intake, but then become worried about the long-term effect of binge eating on their weight. They may then feel prompted, possibly by PU, to engage in behaviors that bring about positive emotions, reduce negative affect about weight gain, *and* promote weight loss (e.g., exercise; Kotbagi et al., 2017), thus maintaining the binge-compensatory behavior cycle; such a possibility merits investigation. Importantly, this risk process may also extend to other eating disorders, such as anorexia nervosa binge-purge type.

Results suggest assessing PU in BN may be helpful in identifying those at-risk for greater binge size and its consequences. Clinicians should consider a stronger focus on negative *and* positive emotions during binge eating treatment. Treatments focused on identifying, accepting, and managing negative and positive emotions, such as integrative cognitive-affective therapy, hold promise for reducing BN symptoms (Wonderlich et al., 2014). Interventions emphasizing mindfulness and skills training to regulate emotions, such as dialectical behavior therapy, also have been shown to decrease binge eating (Ben-Porath et al., 2020). Individuals high in PU may benefit from practicing new ways to respond to positive emotions. If one often responds to extreme positive emotions with binge eating, they may instead try calling a loved one or engaging in a sensory activity (e.g., polishing one’s nails). If individuals high in PU experience positive reinforcement from binge eating, they may find it helpful to identify other, more adaptive strategies to elicit positive emotions, such as building mastery in a new hobby.

Interestingly, it is possible that PU may be helpful in facilitating eating disorder recovery; a recent study found that among eating disorder patients, PU was the only personality positively associated with recovery (Cabelguen et al., 2023). Clinicians may find it useful to capitalize on the high levels of positive emotions that accompany PU and potentially promote recovery.

Study strengths included the use of a well-validated laboratory paradigm for binge eating, inclusion of a clinical sample with BN, and use of measures with strong psychometric properties. Limitations are as follows. Our sample consisted of relatively young women. Additionally, group samples were small, although well-powered to detect differences. This study should be replicated in larger samples that are more diverse in terms of gender and age. We used a tightly-controlled laboratory design to test NU and PU’s prediction of binge size, measured objectively. As such, results may or may not reflect binge consumption in one’s everyday life, in their natural environment that is more familiar and less controlled. We cannot know if results would differ if we assessed positive urgency’s prediction of binge size in one’s daily life, as in an ecological momentary assessment design; future research should test that possibility. Finally, given the correlation between PU and NU, replication of the current study to further confirm the role of PU in predicting binge size, using the same controls, is important.

PU appears to be an underappreciated, but potentially important, predictor of binge size in BN. Clinicians and researchers are encouraged to consider PU in their understanding of binge eating in BN.

## Data availability statement

The raw data supporting the conclusions of this article will be made available by the authors, without undue reservation.

## Ethics statement

The studies involving human participants were reviewed and approved by University of Kentucky Institutional Review Board. The patients/participants provided their written informed consent to participate in this study.

## Author contributions

HD and GS contributed to the conception and design of the study. HD organized the database, performed statistical analyses, and wrote the first draft of the manuscript. All authors contributed to manuscript revision, read, and approved the submitted version.

## Funding

This work was supported by a grant from the National Institute of Mental Health (F31 MH114551). The funder had no role in study design, in the data collection, analysis and interpretation of data, in the writing of the report, or in the decision to submit the article for publication.

## Conflict of interest

The authors declare that the research was conducted in the absence of any commercial or financial relationships that could be construed as a potential conflict of interest.

## Publisher’s note

All claims expressed in this article are solely those of the authors and do not necessarily represent those of their affiliated organizations, or those of the publisher, the editors and the reviewers. Any product that may be evaluated in this article, or claim that may be made by its manufacturer, is not guaranteed or endorsed by the publisher.

## Footnotes

^1^Full study sample included *N* = 51 participants. One control participant was excluded because she had no objective test meal consumption data due to an equipment malfunction.

^2^Four participants in the BN group endorsed fewer than 12 episodes of binge eating in the past 12 weeks (range: 6–11 episodes), thus meeting criteria for OSFED BN. Results did not differ when these participants were excluded from analyses, so they were retained. “BN group” refers to all 21 participants who met criteria for BN or OSFED BN.

^3^Eligibility was determined based on participants’ self-reported height/weight on the phone screen. Upon objective measurement of height/weight, four participants had BMIs over 26.5. Results did not differ when those with higher BMIs were excluded from analyses, so they were retained.

^4^Because state levels of negative affect may influence binge size, we ran our model with state negative affect (measured using a composite of negative emotion ratings; Davis et al., 2022) included as a predictor. It was not a significant predictor of test meal consumption and results did not differ when it was included. Thus, we retained our original model.
